# Thermosensitive Hydrogels for Periodontal Regeneration: A Systematic Review of the Evidence

**DOI:** 10.1002/cre2.70029

**Published:** 2024-11-13

**Authors:** Mohammad Amin Amiri, Delara Amiri, Shahram Hamedani

**Affiliations:** ^1^ Oral and Dental Disease Research Center, School of Dentistry Shiraz University of Medical Sciences Shiraz Iran; ^2^ School of Dentistry Shiraz University of Medical Sciences Shiraz Iran

**Keywords:** periodontal regeneration, periodontitis, smart biomaterials, thermosensitive hydrogels

## Abstract

**Objectives:**

Thermosensitive hydrogels are now among the most commonly used biomaterials in tissue engineering. Due to their unique characteristics, this review aimed to evaluate the suitability of thermosensitive hydrogels in periodontal regeneration.

**Material and Methods:**

PubMed, Scopus, and Web of Science databases were searched until March 25, 2024, to retrieve relevant articles. The eligibility criteria for the included studies were determined by the designed PICO elements. Results from each included study were extracted, focusing on the three main areas: thermosensitivity, cellular characteristics, and in vivo characteristics.

**Results:**

Nineteen studies were included in our study. The thermosensitivity assessment of the hydrogels indicated a range of sol–gel transition times from 40 s to 20 min based on the type of polymers and the fabrication process. The cellular characterization was assessed based on three main cellular behaviors: cellular viability/proliferation, differentiation, and migration. The in vivo characterization was performed based on two main approaches: radiographic and histologic evaluation.

**Conclusions:**

The results indicated that the addition of bioactive agents could enhance the in vivo efficacy of thermosensitive hydrogels in periodontal regeneration through three main areas: antimicrobial, anti‐inflammatory, and regenerative effects.

## 1 Introduction

Periodontal diseases like periodontitis are highly prevalent and destroy structures such as alveolar bone, cementum, and periodontal ligament (PDL) that support the teeth (Albandar 2005; Hajishengallis 2015; Ebersole et al. 2018; Kuboniwa et al. 2017; Tsukasaki et al. 2018; Ammar et al. 2018; Xu et al. 2019). This can eventually lead to tooth loss and complications with chewing, nutrition intake, and esthetics (Xu et al. 2019; Pihlstrom, Michalowicz, and Johnson 2005; Nasajpour et al. 2018; Kebschull, Demmer, and Papapanou 2010; Arimatsu et al. 2014). Conventional treatments focus on controlling inflammation or regenerating periodontal tissues separately; however, novel approaches aim to address both simultaneously for improved outcomes (Xu et al. 2019; Nevins et al. 2003; Polimeni, Xiropaidis, and Wikesjö 2006; Faggion 2014).

Tissue engineering strategies using stem cells and biomaterial scaffolds seem to be promising choices for regenerating damaged periodontium (Ammar et al. 2018; Chen et al. 2010; Ooaku 1989; Chien et al. 2018). In particular, thermosensitive hydrogels that transition from solution to gel at body temperature have emerged as versatile platforms for periodontal regeneration (Quirynen, Teughels, and van Steenberghe 2003; Liu et al. 2011; Mojsoska and Jenssen 2015; Ji et al. 2010; Liu et al. 2021). Chitosan (CS)‐based hydrogels are among the most common types of thermosensitive hydrogels that exhibit satisfactory biocompatibility, easy delivery by injection, and the ability to sustain the release of therapeutic agents, making them promising for treating periodontitis (Xu et al. 2019; Ji et al. 2010; Tatakis and Kumar 2005; Wei et al. 2019; Feng et al. 2017; Li, Yan, et al. 2017; Guerra et al. 2017; Liu et al. 2017; Wang, Malcolm, and Benoit 2017a). However, CS also has some limitations, such as lacking cell adhesion ligands; thus, incorporating growth factors or bioactive molecules is needed to improve cell viability and proliferation (Ammar et al. 2018; Arpornmaeklong, Pripatnanont, and Suwatwirote 2008, 2007; Arpornmaeklong et al. 2021). Besides, several other biomaterials can be used to design thermosensitive hydrogels for periodontal regeneration. In this regard, thermosensitive polymers such as poly lactic‐co‐glycolic acid (PLGA) or polyethylene glycol (PEG) can also facilitate filling complex bone defects by transitioning from liquid to gel upon injection (Wang, Zhang, et al. 2017; Pan, Deng, et al. 2019; Cao et al. 2019; Yan et al. 2015).

The application of potential biomaterials to develop a thermosensitive hydrogel for periodontal regeneration has brought about satisfactory outcomes (Chien et al. 2018; Liu et al. 2021; Arpornmaeklong et al. 2021; Pan, Deng, et al. 2019; Zang et al. 2014; Liu et al. 2022). Moreover, these hydrogels are also used as proper carriers for potential medications and stem cells that can promote antibacterial (Liu et al. 2021, 2022), anti‐inflammatory (Ammar et al. 2018; Chien et al. 2018), and regenerative effects (Chien et al. 2018; Liu et al. 2021, 2022). In this regard, platelet concentrates, mostly known for their high regenerative effects (Farshidfar et al. 2021, 2022), have shown satisfactory outcomes when loaded into CS hydrogels for sustained release (Liu et al. 2022). Anti‐inflammatory drugs like aspirin and growth factors like erythropoietin (EPO) are promising candidates; however, their short half‐lives necessitate repeated administration (Xu et al. 2019). Stem cell sources, such as PDL stem cells (PDLSCs) and induced pluripotent stem cells (iPSCs) show excellent capacity for regeneration when combined with osteoinductive growth factors, including bone morphogenetic protein‐6 (BMP‐6) in 3D hydrogel environments (Chien et al. 2018).

Despite the emerging application of thermosensitive hydrogels in periodontal regeneration, a comprehensive review of their potential and limitations remains lacking. However, these biomaterials have been extensively reviewed in other domains, such as bone (Abdollahi et al. 2023), cartilage regeneration (Zhang, Yu, et al. 2019), and drug delivery (Huang et al. 2019), underscoring their significant potential given their desirable gelation time at physiological temperatures. The increasing significance of these biomaterials, coupled with the growing body of literature on periodontal regeneration, has motivated the authors to undertake a critical and thorough review in this field. Therefore, the objective of this systematic review is to provide a detailed evaluation of the applicability of thermosensitive hydrogels in periodontal regeneration, synthesizing current evidence to enhance our understanding.

## 2 Materials and Methods

### 2.1 Protocol Design

This systematic review is based on the guidelines provided by the Preferred Reporting Items for Systematic Reviews and Meta‐Analyses (PRISMA) Statement 2020 (Page et al. 2021).

### 2.2 Information Sources and Search Strategy

A thorough and academic search was performed in PubMed, Scopus, and Web of Science databases to retrieve articles associated with thermosensitive hydrogels and periodontal regeneration from January 1, 2000, until August 31, 2024. Moreover, a manual search was further performed on the references of the included articles to find articles associated with the aim of this study.

### 2.3 Eligibility Criteria

To select studies that are suitable for our study, we have developed the eligibility criteria based on the four main parts: participants, intervention, comparison, and outcomes (PICO; Table 1). All the in vitro, in vivo, and clinical studies focusing on the development, characterization, and evaluation of the thermosensitive hydrogels (intervention) for the regeneration of periodontal defects (population) were screened (Table 1). The studies comparing the thermosensitive hydrogels in vitro, in vivo, or clinical settings with the negative control group (absence of hydrogel) were included (comparison). The studies lacking a control group in in vitro, in vivo, or clinical assessments were excluded. Concerning the outcomes, the studies assessing the impact of thermosensitive hydrogels for periodontal regeneration were included.

**Table 1:** PICOS question designed for this systematic review.

PICOS	Inclusion criteria	Exclusion criteria
Population	Studies assessing periodontal regeneration	Studies assessing regeneration strategies other than periodontal regeneration
Intervention	Studies evaluating the effect of thermosensitive hydrogels	Studies evaluating the effect of biomaterials other than thermosensitive hydrogels
Comparison	Studies evaluating the effect of thermosensitive hydrogels compared to the control group	Studies without the control group
Outcome	Studies assessing the impact of thermosensitive hydrogels in periodontal regeneration	Studies assessing the impact of thermosensitive hydrogels in areas other than periodontal regeneration
Study design	In vitro, in vivo, and clinical studies	Case reports, narrative reviews, systematic reviews with or without meta‐analysis, letter to the editors, short communications, in vivo studies, ex vivo studies, animal studies, and non‐comparative studies.

### 2.4 Study Selection

The titles and abstracts of the retrieved papers were separately reviewed by the authors (M.A.A. and D.A.) based on the eligibility requirements. The articles that were recovered were further checked for any potential predatory publications. Studies that met the inclusion criteria were added to our systematic review.

### 2.5 Obtaining Data and Storing Data

In order to present the data of the included studies, a data extraction table was used including the type of biomaterials and agents, applied stem cells, in vitro characterization assays, in vivo characterization assays, and outcomes.

The authors (M.A.A. and D.A.) independently reviewed the titles and abstracts of the retrieved papers based on the qualifying requirements. In case of disagreement, the issue was discussed with the other member of the team (S.H.). The retrieved articles were additionally checked for any potential predatory publications. After obtaining the complete texts of the chosen articles, papers that met the inclusion requirements were added to our systematic review.

## 3 Results

### 3.1 Study Characteristics

**Figure 1:**
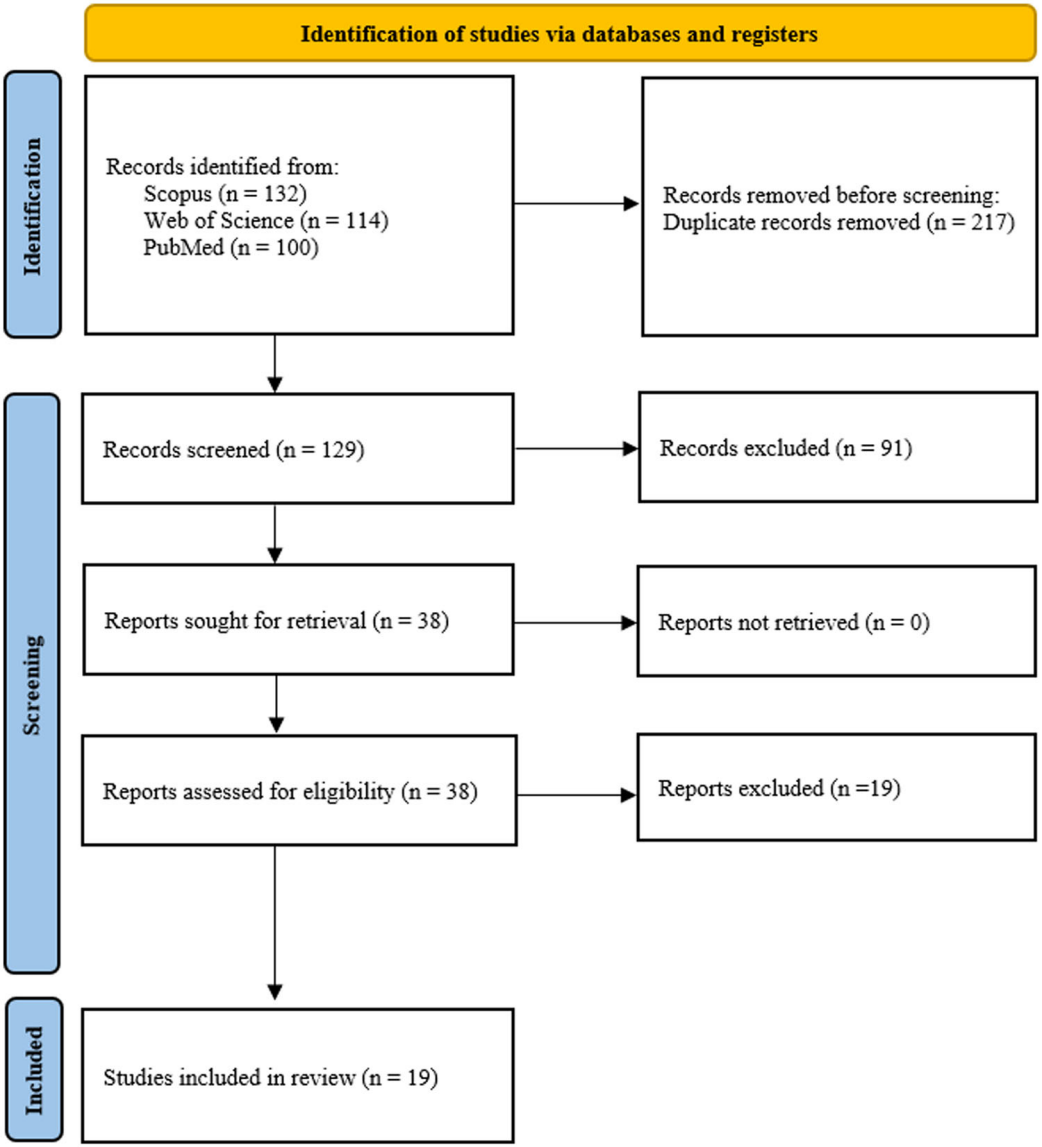
Representation of PRISMA chart 2020 used for the screening process in the present study.

After the primary search on three databases, 346 articles were obtained. After duplicate removal, 129 studies were considered for screening, and 91 studies were excluded due to mismatch. Finally, 38 studies were evaluated based on their titles and abstracts. Nineteen studies were excluded due to a mismatch with the PICO question. Nineteen studies were finally included in this systematic review (Figure 1). Table 2 represents the study characteristics concerning the biomaterials/agents, stem cells, in vitro and in vivo assays, and outcomes.

**Table 2:** Summary of the included studies.

Author (year)	Biomaterials and agents	Stem cells	In vitro characterization	In vivo characterization	Outcomes
Huang et al. (2023)	I. Chitosan II. PVA	—	− SEM − FTIR − TGA − Swelling ratio − Biodegradability − Tensile strength − Cell viability	—	− The PVA/chitosan hydrogel could enhance cell viability.
Tan et al. (2023)	I. β‐TCP II. Chitosan III. β‐GP	—	− SEM − EDX − XRD − FTIR − Rheology − Cell viability and proliferation − Degradability − AlamarBlue assay − Live/dead staining	—	− The application of β‐TCP in chitosan hydrogels seems promising for periodontal bone and soft tissue repair.
Wang, Zhang, et al. (2023)	I. BMP‐2 II. T8IC III. Chitosan IV. Gelatin V. β‐glutamine	—	− Cell viability − Photothermal effects on the cell proliferation − Antibacterial effect − Osteogenic effect − ROS generation	− Toxicity and metabolism of hydrogel − NIR II fluorescence scanner − μ‐CT − Laser irradiation − Osteogenic and anti‐inflammatory effects	− Thermosensitive hydrogels with T8IC, laser, and BMP‐2, H_2_O_2_ showed an excellent bactericidal effect, along with osteogenic induction.
Wang, Liu, et al. (2023)	I. Chitosan II. β‐GP III. CS IV. SA V. Berberine	—	− Cytotoxicity assay − Cell viability − CCK‐8 − Live‐dead cell staining − Anti‐inflammatory effect assay − qRT‐PCR − Western blot analysis − Osteogenic effect assay − ALP staining	—	− The berberine thermosensitive hydrogel is a safe carrier with anti‐inflammatory and osteogenic effects on periodontitis through the PI3K/AKT pathway.
Wang, Chang, et al. (2023)	I. MSN II. Metformin III. PEG IV. PDLLA V. SDF‐1	—	− Hydrogel thermosensitivity − HNMR − FTIR − TEM − Pore volume − Drug release − Fluorescent imaging − RT‐qPCR − Osteogenic differentiation − Intracellular ROS − ALP activity − Alizarin red S Staining − Transwell migration assay − Drug cytotoxicity − Western blot	− Hydrogel degradation − Hydrogel biocompatibility − H&E staining − Masson trichrome staining − Confocal microscopy for rBMSCs migration assessment	− This thermosensitive hydrogel was able to restore the migration and osteogenic capacity of rBMSCs under high glucose concentrations. In in vivo conditions with diabetes type 2, this hydrogel could promote bone regeneration and recruit rBMSCs to the defect area.
Wang, Peng, et al. (2023)	I. DSPE‐PEG‐FA II. Genistein III. Lecitin IV. Cholesterol	—	− SEM − Rheology − Cytotoxicity − Anti‐inflammation − Macrophage polarization − Immunofluorescence staining − Osteogenic differentiation	− μ‐CT − H&E staining − Masson trichrome staining − Immunofluorescence staining − TNF‐α − IL‐1β − IL‐6 − IL‐17	− The FA‐GEN‐Lip@Gel was able to induce a phenotypic change of macrophages from M1 to M2. Moreover, this hydrogel was able to induce anti‐inflammation as well as bone regeneration.
Liu et al. (2022)	I. PEG‐DA II. DTT III. SDF‐1	—	− Cell viability − Intracellular ROS − Mitochondrial ROS − MDA concentration − GSH proportion − Osteogenic differentiation − Cell immunofluorescence staining	− μ‐CT − H&E staining − IHC staining (ALP activity)	− The designed hydrogel could markedly decrease the intracellular ROS and enhance osteogenic differentiation of osteoblasts in an inflamed environment. − The combination of DTT and SDF could induce bone regeneration in periodontitis.
Arpornmaeklong et al. (2021)	I. Chitosan II. Collagen III. Quercetin IV. β‐GP	—	− Degradability − Swelling − Compressive strength − SEM − XRD − FTIR − Quercetin release: 1. UV‐Vis spectrophotometry 2. Mass spectrophotometry 3. TFC assay − Antioxidant assay: 1. DPPH radical scavenging assay 2. Total antioxidant assay − Intracellular ROS assay − Cell viability	—	− Quercetin/β‐GP/chitosan/collagen hydrogel with the quercetin/β‐GP weight ratio of 2:1 demonstrated to have the optimal properties for bone regeneration and sustained release of quercetin.
Liu et al. (2021)	I. PEG‐DA II. DTT III. SDF‐1 IV. FPM V. SAMP	—	− SEM − FTIR − Degradation and Swelling − Release assay − Cell proliferation − Cell migration − Osteogenic differentiation − Antibacterial activity	− μ‐CT − H&E staining − IHC (TNF‐α and IL‐1β) − Immunofluorescence staining (CD90 and CD34)	− The designed hydrogel was able to exert antibacterial activity in the presence of *Porphyromonas gingivalis*, promote a low‐level inflammatory state, accelerate the recruitment of CD90+/CD34− stromal cells, and promote osteogenesis.
Petit et al. (2020)	I. Chitosan II. GP III. Atorvastatin IV. Lovastatin	—	− TEM − Zeta potential − Pore size and porosity − Release activity − Cell metabolic activity − Nanoemulsion cellular uptake − RT‐PCR − Immunofluorescence staining	− Alizarin red staining − Immunofluorescence staining	− The atorvastatin/lovastatin nanoemulsion loaded Chitosan/GP hydrogel could decrease the level of inflammation caused by *P. gingivalis* and enhance the osteogenic ability of osteoblasts. In an in vivo setting, this hydrogel significantly enhanced bone formation compared to the systemic administration of statin drugs.
Pan, Deng, et al. (2019)	I. PLGA II. PEG	PDLSCs	− Alizarin red S staining − Oil red O staining − Cell viability	− μ‐CT − H&E staining − Masson's trichrome staining − IHC	− The PLGA/PEG hydrogel was an effective environment for cell culture. The incorporation of PDLSC into PLGA/PEG hydrogel resulted in the overexpression of PDGF‐BB.
Xu et al. (2019)	I. Chitosan II. Gelatin III. β‐GP IV. Aspirin V. EPO	—	− Rheology − SEM − In vitro aspirin/EPO release − Cell viability	− In vivo biocompatibility (subcutaneous implantation) − μ‐CT − IHC	− The chitosan/gelatin/β‐GP hydrogel loaded with aspirin and EPO indicated the faster release of aspirin compared to EPO. This hydrogel could decrease periodontal inflammation and aid in recovering the alveolar bone.
Zang, Mu, et al. (2019)	I. Chitosan II. β‐GP III. BMP‐7 IV. ORN	—	− Hydrogel thermosensitivity − SEM − Viscosity − Swelling − Biodegradation − Drug release − Antimicrobial assay	− H&E staining − Masson's trichrome staining − TRAP staining − OCN staining	− Chitosan/β‐GP/BMP‐7/ORN hydrogel exhibited sustained release patterns for ORN and BMP‐7. Besides, ORN showed a significant antibacterial effect against *P. gingivalis* The histological results indicated significant bone regeneration and less connective in class III furcation defects when BMP‐7 was applied.
Ammar et al. (2018)	I. Chitosan II. Quaternized chitosan III. β‐GP IV. FDPC	PDLSCs	− Thermosensitivity − GF release − Cell viability	—	− FDPC‐loaded hydrogels exhibited sustained release of TGF‐β1 and PDGF‐BB for 2 weeks. − FDPC didn't affect sol–gel transition and enhanced PDLSC viability.
Chien et al. (2018)	I. Chitosan II. Gelatin III. β‐GP IV. BMP‐6	i‐PSCs	− Cell viability − Osteogenic differentiation − Alizarin red S staining	− μ‐CT − H&E staining	− iPSCs and BMP‐6 showed synergistic effects on bone and cementum regeneration. The addition of both iPSCs and BMP‐6 to chitosan/gelatin/β‐GP hydrogel contributed to both anti‐inflammation and periodontal regeneration.
Li, Pan, et al. (2017)	I. Chitosan II. GP III. BMP‐2 plasmid DNA IV. Chitosan nanoparticles	—	− Hydrogel thermosensitivity − SEM − Swelling − Degradation − In vitro DNA release − Cell viability − Cell proliferation	—	− The addition of chitosan nanoparticle‐plasmid DNA‐BMP‐2 to chitosan/GP enhanced cell growth and exerted proper cytocompatibility. This thermosensitive hydrogel was suggested as a suitable candidate for gene delivery.
Li, Ji, et al. (2017)	I. Chitosan II. GP III. BMP‐2 plasmid DNA IV. Chitosan nanoparticles	—	− Hydrogel thermosensitivity − SEM	− Masson's trichrome staining − ALP staining	− The chitosan/GP/chitosan nanoparticle/BMP‐2 plasmid DNA thermosensitive hydrogel induced proper bone regeneration in periodontitis model with non‐specific inflammatory reaction.
Zang et al. (2014)	I. Chitosan II. β‐GP	—	− Thermosensitivity − SEM − Pore size − Swelling ratio − Biodegradability − Cell viability − Cell adhesion	Van Gieson's staining	− The comparison of chitosan powder and solution when preparing the chitosan/β‐GP hydrogel, showed chitosan powder shortened gelation time, enhanced viscosity and water absorption, and had acceptable degradability, porosity, and cell viability.
Ji et al. (2010)	I. Chitosan II. Quaternized chitosan III. β‐GP	—	− Hydrogel thermosensitivity − Cell viability and morphology − ALP activity	Mallory's trichrome staining	The chitosan/HTCC/β‐GP exerted acceptable biocompatibility and when incorporated with bFGF, it enhanced periodontal regeneration effectively.

Abbreviations: ALP, alkaline phosphatase; β‐GP, β‐glyceolphosphate; BMP‐7, bone morphogenetic protein‐7; CS, chitosan; DLS, dynamic light scattering; DPPH, 2,2‐Diphenyl‐1‐picrylhydrazyl; DTT, dithiothreitol; EDX, energy dispersive X‐ray analysis; EPO, erythropoietin; FDPC, freeze‐dried platelet concentrate; FTIR, fourier transform infra‐red spectroscopy, HNMR: proton nuclear magnetic resonance spectroscopy; H & E, hematoxylin and eosin; IHC, immunohistochemistry; IL, interleukin; iPSC, induced pluripotent stem cell; MDA, malondialdehyde; µ‐CT: micro‐computed tomography; ORN, ornidazole; PDLSCs, periodontal ligament stem cells; PEG, polyethylene glycol; PLGA, poly lactic‐co‐glycolic acid; rBMSCs, rat bone marrow stem cell; ROS, reactive oxygen species; RT‐qPCR, reverse transcriptase quantitative polymerase chain reaction; SA, sodium alginate; SDF1, stromal cell‐derived factor‐1; SEM, scanning electron microscopy; TEM, transmission electron microscopy; TGA, thermogravimetric analysis; TNF‐α, transforming growth factor‐α, XRD, X‐Ray diffraction analysis.

Based on the included studies, CS was used in 13 studies to design a thermosensitive hydrogel (Ammar et al. 2018; Xu et al. 2019; Li, Ji, et al. 2017; Wang, Zhang, et al. 2023; Tan et al. 2023; Chien et al. 2018; Ji et al. 2010; Arpornmaeklong et al. 2021; Zang et al. 2014; Huang et al. 2023; Petit et al. 2020; Wang, Peng, et al. 2023; Li et al. 2016). However, quaternized CS was used in two studies combined with CS and β‐glycerol phosphate (β‐GP) (Ammar et al. 2018; Ji et al. 2010). GP was also utilized in 12 studies (Ammar et al. 2018; Xu et al. 2019; Zang, Yu, et al. 2019; Chien et al. 2018; Wang, Liu, et al. 2023; Chien et al. 2018; Ji et al. 2010; Arpornmaeklong et al. 2021; Zang et al. 2014; Petit et al. 2020; Li et al. 2016; Li, Ji, et al. 2017; Tan et al. 2023) out of which, eight studies specifically reported that they used β‐GP (Ammar et al. 2018; Xu et al. 2019; Chien et al. 2018; Ji et al. 2010; Arpornmaeklong et al. 2021; Tan et al. 2023; Zang, Mu, et al. 2019; Wang, Liu, et al. 2023). As for the other types of natural biomaterials, it was further indicated that collagen and gelatin were used in one (Arpornmaeklong et al. 2021) and two studies (Xu et al. 2019; Chien et al. 2018), respectively.

Concerning the application of synthetic biomaterials, seven studies have incorporated these biomaterials to develop thermosensitive hydrogels (Ammar et al. 2018; Xu et al. 2019; Chien et al. 2018; Ji et al. 2010; Arpornmaeklong et al. 2021; Zang et al. 2014; Huang et al. 2023). Among these biomaterials, PLGA, PEG, and polyvinyl alcohol (PVA) were used (Liu et al. 2021; Pan, Deng, et al. 2019; Liu et al. 2022; Huang et al. 2023; Wang, Peng et al. 2023; Wang, Chang, et al. 2023).

Moreover, 17 studies have not loaded stem cells in their hydrogels (Xu et al. 2019; Ji et al. 2010; Li, Ji, et al. 2017; Wang, Zhang, et al. 2023; Tan et al. 2023; Zang, Yu, et al. 2019; Wang, Liu, et al. 2023; Wang, Chang, et al. 2023; Liu et al. 2021; Arpornmaeklong et al. 2021; Zang et al. 2014; Liu et al. 2022; Huang et al. 2023; Petit et al. 2020; Wang, Peng, et al. 2023; Li et al. 2016); however, two studies have incorporated PDLSCs (Ammar et al. 2018; Pan, Deng, et al. 2019), and one study has applied iPSCs (Chien et al. 2018) for periodontal regeneration.

Various bioactive agents and medications were included in the final composition of the hydrogels in the studies (Ammar et al. 2018; Chien et al. 2018; Liu et al. 2021; Arpornmaeklong et al. 2021; Liu et al. 2022; Petit et al. 2020; Zang, Mu, et al. 2019). In this regard, seven studies have applied bioactive agents and medications, including aspirin (Xu et al. 2019), atorvastatin (Petit et al. 2020), BMP‐6 (Chien et al. 2018), BMP‐7 (Wang, Zhang, et al. 2023; Zang, Mu, et al. 2019), dithiothreitol (DTT) (Liu et al. 2021), EPO (Xu et al. 2019), freeze‐dried platelet concentrate (FDPC) (Ammar et al. 2018), functional peptide module (FPM) (Liu et al. 2021), genistein (Wang, Peng, et al. 2023), lovastatin (Petit et al. 2020), ornidazole (ORN) (Zang, Mu, et al. 2019), plasma DNA (pDNA) of BMP‐2 (Li et al. 2016; Li, Ji, et al. 2017), short antimicrobial peptide (SAMP) (Liu et al. 2021), stromal cell‐derived factor‐1 (SDF‐1) (Liu et al. 2021; Wang, Chang, et al. 2023), and quercetin (Arpornmaeklong et al. 2021).

Concerning the characterization methods, all the studies have conducted in vitro characterization methods; however, only seven studies have carried out in vivo characterizations.

## 4 In Vitro Thermosensitivity Characterization

Out of the 19 included studies, two studies have reported that the sol–gel transition of their designed thermosensitive hydrogels occurred during 10 min at 37°C (Ammar et al. 2018; Arpornmaeklong et al. 2021). In the study by Arpornmaeklong et al. (2021), it was demonstrated that in the thermosensitive hydrogel of CS/collagen/β‐GP, the sol–gel transition could vary from 10 to 20 min at 37°C. This spectrum is dependent upon the content of collagen in the final solution as the collagen itself exhibited 20 min at 37°C, whereas in a 1:1 proportion of CS:collagen, the sol–gel transition could reduce to 10 min.

In a study using CS/gelatin/β‐GP (Xu et al. 2019), the sol–gel transition at 37°C was 5 min. In one study designing the CS/β‐GP, it was demonstrated that, initially, if the CS is autoclaved after being dissolved in 0.1 mol/L of hydrochloric acid, the final CS/β‐GP will have a sol–gel transition rate of 30 min at 37°C. Nevertheless, if the CS is autoclaved before being dissolved in 0.1 mol/L of hydrochloric acid, it would take 5–6 min for the final CS/β‐GP to transform into a gel.

Three studies reported a sol–gel transition time of 3 min at 37°C for thermosensitive hydrogels, including CS/quaternized CS (HTCC)/GP (Ji et al. 2010) and CS nanoparticle (CSn)—α,β‐GP (Li et al. 2016; Li, Ji, et al. 2017).

The results of studies conducted by Li et al. (2016) Li, Ji, et al (2017) indicated that when adding CSn to the CS/α,β‐GP, the thermogelling time decreases from 5 to 3 min. This is probably attributed to CSn facilitating CS polymer interactions and polymerization at 37°C. Moreover, adding pDNA‐BMP‐2 to CSn in the mentioned hydrogel was reported to not affect the thermosensitivity characteristics (Li et al. 2016; Li, Ji, et al. 2017).

The most rapid sol–gel transition among the studies was 40 s at 37°C (Wang, Peng, et al. 2023). The thermosensitive hydrogel was folic acid–modified liposome hydrogel loaded with genistein (FA‐GEN‐Lip@Gel). The hydrogel was based on genistein, lecithin, cholesterol, and DSPE‐PEG‐FA.

## 5 In Vitro Cellular Characterization

Concerning the cellular characterization of thermosensitive hydrogels, all of the 19 studies have assessed cell viability except for two studies (Li, Ji, et al. 2017; Zang, Mu, et al. 2019). The most frequently used cell for the assessment of cell viability was PDLSCs (Ammar et al. 2018; Ji et al. 2010; Liu et al. 2021; Arpornmaeklong et al. 2021; Pan, Deng, et al. 2019; Zang et al. 2014; Wang, Peng, et al. 2023; Li et al. 2016). Besides, the most common technique of the assessment of cell viability was MTT (3‐[4,5‐dimethylthiazol‐2‐yl]‐2,5 diphenyl tetrazolium bromide) assay (Ammar et al. 2018; Xu et al. 2019; Chien et al. 2018; Ji et al. 2010; Zang et al. 2014; Huang et al. 2023). All the studies have reported acceptable biocompatibility for their hydrogels (Xu et al. 2019; Chien et al. 2018; Wang, Chang, et al. 2023; Ji et al. 2010; Liu et al. 2021; Pan, Deng, et al. 2019; Liu et al. 2022; Wang, Peng, et al. 2023; Wang, Zang et al 2023; Tan et al. 2023; Wang, Liu, et al. 2023). Besides, the studies that have incorporated a bioactive agent into the thermosensitive hydrogels have exhibited significant outcomes (Ammar et al. 2018; Xu et al. 2019; Liu et al. 2021; Pan, Deng, et al. 2019; Petit et al. 2020; Zang, Mu, et al. 2019); in this regard, the addition of FDPC (Ammar et al. 2018), quercetin, and the combination of SDF‐1 and DTT have shown remarkable outcomes in enhancement of cell viability and proliferation on thermosensitive hydrogels (Liu et al. 2021).

Concerning other methods of studying cellular behavior on thermosensitive hydrogels, six studies have assessed osteogenic differentiation via alkaline phosphatase (ALP) activity, ALP staining, and alizarin red S staining (Ji et al. 2010; Liu et al. 2021, 2022; Wang, Peng, et al. 2023; Wamg, Liu, et al. 2023; Wang, Chang, et al. 2023). In the study by Liu et al. (2022), it was found that although the bacterial lipopolysaccharides (LPS) can reduce the ALP activity of osteoblasts, the PEGD@SDF‐1 thermosensitive hydrogel could almost restore the osteoblasts' ALP activity to the normal level. In the study by Ji et al. (2010), it was demonstrated that the CS/HTCC/GP thermosensitive hydrogel can exhibit positive effects on the enhancement of ALP activity of PDLSCs; however, this effect did not endure the 5 days of the study (Ji et al. 2010). Moreover, the study by Liu et al. (2021) indicated that PEGD@SDF‐1 and PEGPD@SDF‐1 thermosensitive hydrogels exerted the desirable outcomes concerning the ALP activity and mineralization activity (Alizarin red S staining) of PDLSCs.

Among the other tests utilized to assess the cellular activity of hydrogels, two studies assessed the migration activity (Liu et al. 2021; Wang, Chang, et al. 2023). In the study by Liu et al. (2021), PDLSCs were tested for migration using a transwell migration assay. It was found that PEGD@SDF‐1 and PEGPD@SDF‐1 thermosensitive hydrogels exhibited the highest rate of cell migration compared to the PEGD, and PEGPD hydrogels, and the negative control group (Liu et al. 2021). In the study by Wang, Chang, et al. (2023), a thermosensitive hydrogel consisting of PDLLA (poly(d, l‐lactic acid) and PPP (PDLLA‐PEG‐PDLLA) was designed. To enhance the biological impact, metformin, mesoporous silica, and SDF‐1 were incorporated (Wang, Chang, et al. 2023). The results indicated that when the migration ability of bone marrow mesenchymal stromal cells (BMSCs) was impaired due to high glucose concentration (44 mM), the addition of this hydrogel could restore the migration ability of BMSCs (Wang, Chang, et al. 2023).

## 6 In Vivo Characterization

Among the 19 included studies, eight studies did not evaluate the in vivo behavior of the thermosensitive hydrogels (Ammar et al. 2018; Arpornmaeklong et al. 2021; Huang et al. 2023; Li et al. 2016; Wang, Zhang, et al. 2023; Tan et al. 2023; Wang, Liu, et al. 2023). Out of the 12 studies conducting in vivo characterization, eight studies (Xu et al. 2019; Chien et al. 2018; Ji et al. 2010; Liu et al. 2021; Pan, Deng, et al. 2019; Liu et al. 2022; Wang, Peng, et al. 2023; Wang, Chang, et al. 2023) have utilized micro‐computed tomography (μ‐CT) to assess the study outcomes, one study used the conventional radiography method (Zang, Mu, et al. 2019), and three studies did not apply any radiographic examinations (Zang et al. 2014; Petit et al. 2020; Li, Ji, et al. 2017). On the other hand, all 12 studies (Xu et al. 2019; Chien et al. 2018; Zang, Mu, et al. 2019; Wang, Chang, et al. 2023; Ji et al. 2010; Liu et al. 2021; Pan, Deng, et al. 2019; Zang et al. 2014; Liu et al. 2022; Petit et al. 2020; Wang, Peng, et al. 2023; Li, Ji, et al. 2017) have conducted histological examinations to assess their in vivo results. Among the histology techniques, hematoxylin and eosin (H&E) staining were the most commonly used approach in all the studies (Xu et al. 2019; Chien et al. 2018; Wang, Chang, et al. 2023; Ji et al. 2010; Liu et al. 2021; Pan, Deng, et al. 2019; Zang et al. 2014; Liu et al. 2022; Wang, Peng, et al. 2023; Li, Ji, et al. 2017; Zang, Mu, et al. 2019), except Zang et al. (2014), which have only applied Van Gieson's staining, and Petit et al. (2020), utilizing alizarin red and immunofluorescence calcein staining methods. The results of studies have indicated that the incorporation of stem cells (iPSCs), anti‐inflammatory agents (aspirin, iPSCs, and SDF‐1), antibacterial agents (SAMP), and osteoinductive agents (EPO, BMP‐6, PDLSCs, platelet‐derived growth factor‐BB [PDGF‐BB], and SDF‐1) can highly influence the clinical impact of thermosensitive hydrogels in in vivo periodontitis models.

## 7 Discussion

**Figure 2:**
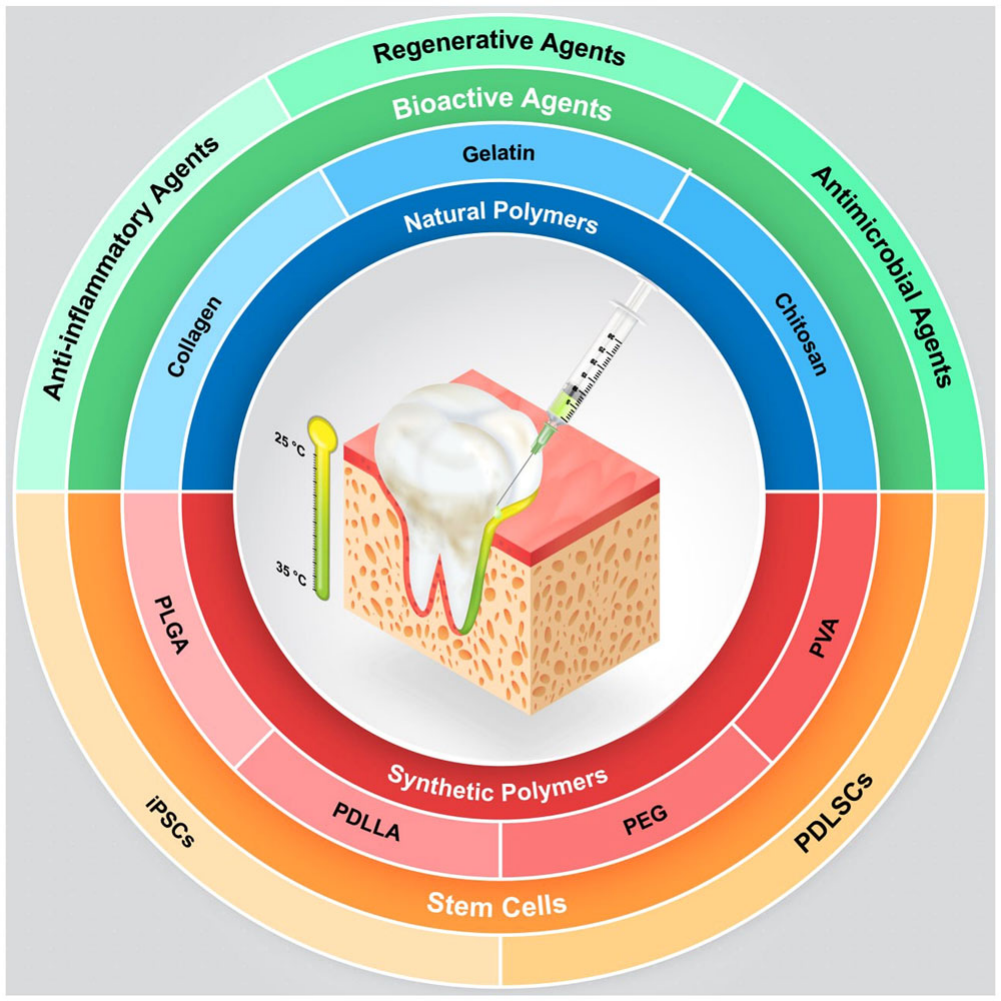
Schematic representation of the thermal changes during the application of thermosensitive hydrogels for periodontal regeneration. The currently used polymers in this regard are categorized as natural and synthetic. Natural polymers include collagen, gelatin, and chitosan, whereas synthetic polymers include PLGA, PDLLA, PEG, and PVA. The currently used bioactive agents are categorized as anti‐inflammatory, regenerative, and antimicrobial agents. Moreover, there are only two types of stem cells used in thermosensitive hydrogels for periodontal regeneration: iPSCs and PDLSCs.

Nowadays, thermosensitive hydrogels are considered one of the potential candidates in regenerative medicine. The advantage of these smart biomaterials is their gelation process is adjusted with the body temperature. This could give the advantage of transition from solution to gel state when the temperature of the environment reaches the sol–gel transition temperature. The application of thermosensitive hydrogels in periodontal regeneration is schematically illustrated in Figure 2 with specifications on the types of applied polymers, bioactive agents, and stem cells.

Concerning the applicability of thermosensitive hydrogels in periodontal regeneration, significant outcomes were found (Ammar et al. 2018; Xu et al. 2019; Chien et al. 2018; Ji et al. 2010; Liu et al. 2021; Arpornmaeklong et al. 2021; Pan, Deng, et al. 2019; Zang et al. 2014; Liu et al. 2022; Huang et al. 2023). The basic biomaterials used to design these hydrogels were mainly CS–GP (Ammar et al. 2018; Xu et al. 2019; Chien et al. 2018; Ji et al. 2010; Arpornmaeklong et al. 2021; Zang et al. 2014) or PEG–PLGA (Liu et al. 2021; Pan, Deng, et al. 2019; Liu et al. 2022). The composition of CS/β‐GP was the most commonly used composition to manufacture thermosensitive hydrogels in periodontal regeneration (Ammar et al. 2018; Xu et al. 2019; Chien et al. 2018; Ji et al. 2010; Arpornmaeklong et al. 2021; Zang et al. 2014). CS does not exhibit any thermosensitive behavior alone (Fan et al. 2022); however, when incorporated with sodium GP, the amino groups of the positively charged chains of CS develop an electrostatic bond with the phosphate group of GP (Fan et al. 2022). GP is known to play a catalyst role in the sol–gel transition of CS–GP hydrogels at physiological pH and temperature (Rahmanian‐Devin, Baradaran Rahimi, and Askari 2021). As the temperature rises, the electrical bonds disappear and the CS chains become devoid of water molecules, which lets the hydrophobic interactions increase between the CS chains (Rahmanian‐Devin, Baradaran Rahimi, and Askari 2021). This process is undertaken when sol–gel transition occurs. The temperature of the sol–gel transition of CS–GP can be adjusted through several factors, including CS's molecular weight (Zhou et al. 2008), CSs degree of deacetylation (Rahmanian‐Devin, Baradaran Rahimi, and Askari 2021; Zhou et al. 2008; Ruel‐Gariépy et al. 2000), pH of the solution (Rahmanian‐Devin, Baradaran Rahimi, and Askari 2021; Wu, Su, and Ma 2006), and the concentration of GP (Rahmanian‐Devin, Baradaran Rahimi, and Askari 2021; Wu, Su, and Ma 2006). The other commonly used biomaterial for developing thermosensitive hydrogels for periodontal regeneration was PEG. A common way of utilizing this biomaterial to design thermosensitive hydrogels is the fabrication of PLGA–PEG–PLGA triblock structure (Fan et al. 2022). This polymer has a core–shell structure, with a hydrophilic PLGA shell and a hydrophobic PEG core (Fan et al. 2022). During the phase transition, the hydrophobic interactions between the PEG polymers strengthen and the PLGA shells become more dehydrated (Yu et al. 2009; Yuan et al. 2022). This process would lead to the aggregation of PLGA–PEG–PLGA spherical micelles (Yu et al. 2009; Yuan et al. 2022). The natural and synthetic polymers used to develop thermosensitive hydrogels are represented in Figure 2. Among the applied polymers, CS was the most commonly used biomaterial.

To enhance the efficacy of thermosensitive hydrogels, various bioactive agents, and stem cells have been incorporated into these biomaterials. A summary of the included bioactive agents and stem cells with their intended impact is represented in Figure 2. Based on the nature of periodontal diseases, the added bioactive agents to hydrogels should mainly be involved in either of the following three activities: antibacterial activity, anti‐inflammatory activity, and regenerative effects (Figure 2).

Concerning the addition of medications with anti‐inflammatory activities, in the study by Xu et al. (2019), aspirin and EPO were both added to CS/gelatin/β‐GP thermosensitive hydrogel, which was applied to a periodontitis‐induced model. It was indicated that the main advantage of adding aspirin was to decrease inflammation by inhibiting cyclooxygenase‐2 (COX‐2) and matrix metalloproteinase‐9 (MMP‐9) (Xu et al. 2019). Besides, EPO did not exhibit effective anti‐inflammatory effects, it could enhance the rate of bone regeneration. The regenerative effects of EPO in bone are attributed to both osteogenic and angiogenic effects (Shiozawa et al. 2010; Bretz et al. 2018; Heeschen et al. 2003; Zwezdaryk et al. 2007). EPO is demonstrated to induce osteoblast differentiation, as well as indirect inhibition of osteoclast function through the ephrinB2‐EphB4 signaling pathway (Li et al. 2015). Besides, it is important to note that the medication release rate by CS thermosensitive hydrogel is dependent upon the degree of hydrophobicity of the loaded medication because in CS‐based thermosensitive hydrogels, hydrophobic interaction of CS chains is a critical factor in the gelation process (Fan et al. 2022; Rahmanian‐Devin, Baradaran Rahimi, and Askari 2021). Therefore, the higher hydrophobicity of the medication predicts its slower release in a longer period, whereas, for more hydrophilic medications, a more rapid release rate is expected (Fan et al. 2022; Rahmanian‐Devin, Baradaran Rahimi, and Askari 2021; Qin et al. 2018). This idea could explain the higher release rate of aspirin compared to EPO in the study by Xu et al. (2019) because EPO is more hydrophobic (Toyoda, Arakawa, and Yamaguchi 2002).

Furthermore, in the study conducted by Wang, Peng, et al. (2023), the anti‐inflammatory effect of a tyrosine kinase inhibitor known as genistein in combination with a folic‐acid‐liposome hydrogel (FA‐GEN‐Lip@Gel), consisting of lecithin, cholesterol, and DSPE‐PEG_2000_‐FA, was assessed. According to the in vivo and in vitro assessments, genistein changes macrophages' phenotypes from M1 to M2 (anti‐inflammatory type) by blocking the TLR4/MyD88/NF‐κB pathway. Genistein consequently promotes the release of IL‐10, which is an anti‐inflammatory cytokine, while decreasing the release of inflammatory mediators such as IL‐6, IL‐17, and TNF‐β. It is worth mentioning that as an indirect outcome of the subsided inflammation and dropped expression of IL‐6 and TNF‐β, which block the osteoblast differentiation (Pan, Wang, and Chen 2019), genistein could induce osteogenic differentiation of PDLSCs, thereby acting as a potential regenerative material.

Concerning the addition of medications with regenerative activities, in a study by Chien et al. (2018), BMP‐6 was loaded on a CS/gelatin/β‐GP hydrogel. However, the addition of BMP‐6 did not seem to have a significant impact on the new bone volume, trabecular number, and trabecular thickness compared to the hydrogel alone in the radiographic examination (Chien et al. 2018). Furthermore, the immunostaining results indicated that there was no difference between CS/gelatin/β‐GP and CS/gelatin/β‐GP/BMP‐6 hydrogel groups in the number of ALP (+) and TRAP (+) cells as well as the IL‐8, IL‐1β, and TNF‐α staining (Chien et al. 2018). However, in the histologic evaluation, it was found that the addition of BMP‐6 to CS/gelatin/β‐GP hydrogel could further enhance the new connective tissue formation besides new bone formation (Chien et al. 2018).

Regarding the regenerative activity, in the study by Zang, Mu, et al. (2019), in vitro and in vivo assessments of the regenerative effect of BMP‐7 in combination with CS/β‐GP with or without ORN in class III furcation defects were carried out. Radiographic investigation revealed a comprehensive regeneration of bone along the entire defect 8 weeks following the application of BMP‐7 (Zang, Mu, et al. 2019). Meanwhile, histologic evaluation showed a thorough formation of new bone, cementum, and PDL all along the notch to the fornix of the furcation in the presence of BMP‐7, whereas such an exhaustive manner of regeneration throughout the whole defective area was not detected in the absence of BMP‐7 (Zang, Mu, et al. 2019). Besides, according to TRAP and OCN staining, it was clarified that both BMP‐7 and ORN could contribute to a decreased number of osteoclasts individually; however, the number of osteoblasts was merely increased by BMP‐7 (Zang, Mu, et al. 2019).

To evaluate the regenerative effect of pDNA‐BMP2 when added to CS–CSn–GP, in the study by Li, Ji, et al. (2017), rat calvarial defects and periodontitis model of beagle dog were implanted with CS–GP and CS–CSn (pDNA‐BMP2)–GP and compared with control groups without hydrogels. Despite the limited regeneration potency of calvarial bone (Blanquaert et al. 1995), H&E staining of the rat defects showed new bone formation 4 weeks after implantation with pDNA‐BMP2, which became mature 8 weeks post‐implantation. Although some areas of sporadic bone formation were also seen in the CS–GP group, it seems that the addition of pDNA‐BMP2 could lead to a notably enhanced bone quality and quantity. Regarding the periodontitis model of the beagle dog, Masson's staining showed a higher degree of bone mineralization in CS–CSn (pDNA‐BMP2)–GP group compared to CS–GP group, and ALP assay revealed ALP activity (an osteoblast‐differentiation marker) to be the highest among CS–CSn (pDNA‐BMP2)–GP compared to CS/GP and control groups, indicating the endogenous bone regeneration potential of pDNA‐BMP2 (Li, Ji, et al. 2017).

Another bioactive material loaded on CS–β‐GP to promote periodontal regeneration was FDPC (Ammar et al. 2018). The addition of FDPC did not affect the sol–gel transition characteristics; however, the initial viscosity of the hydrogel appeared to decrease. This was ascribed to the possibility of the positive charges of growth factors within FDPC interacting with the positive amine charges of CS (Ammar et al. 2018; Yamamoto et al. 2000; Chen, and He 2013; Chen, Lewallen, and Xie 2013; King and Krebsbach 2012; Zhao, Zhou, and Wang 2015). In this manner, the repulsion forces within the hydrogel are enhanced resulting in lower viscosity and internal integrity of the hydrogel (Ammar et al. 2018). The addition of FDPC could accelerate the process of periodontal regeneration through the release of various growth factors, including transforming growth factor‐β (TGF‐β), insulin‐like growth factor‐1 (IGF‐1), and PDGF‐BB (Ammar et al. 2018). The TGF‐β and IGF‐1 facilitated the extracellular matrix formation (Jeong Park et al. 2000). Moreover, TGF‐β can promote enhanced cell proliferation and survival rate (Huang and Huang 2005; Moustakas et al. 2002). The PDGF‐BB is considered an effective mitogenic factor and chemo‐attractant for mesenchymal stem cells, especially periodontal progenitor cells (Raja, Byakod, and Pudakalkatti 2009; Javed et al. 2011).

In the study by Wang, Chang, et al. (2023), the stimulating effect of PDLLA‐PEG‐PDLLA‐Met@MSN‐SDF‐1 (PPP‐MM‐S) hydrogel on diabetic periodontal bone regeneration was evaluated. According to the results, in high glucose in vitro environment, metformin eliminated the overproduced reactive oxygen species (ROS) and partially reactivated the AMPK/β‐catenin pathway in rBMSCs, therefore restoring the osteogenic differentiation of rBMSCs significantly. However, in a normal glucose environment, this pathway, and accordingly osteogenesis, did not seem to be triggered by metformin drastically (Wang, Chang, et al. 2023). Additionally, SDF‐1 application can restore the impaired migration ability of rBMSCs, weakened by high glucose, thereby enhancing bone regeneration. In vivo assessment of periodontal bone defects in type 2 diabetes (T2DM) rats showed that rapid diffusion of SDF‐1, a key signal in the recruitment‐osteogenesis cascade, enhanced rBMSC migration to the defect site. Regarding the regenerative capacity, the prolonged releases of metformin up to 4 weeks in the group treated with PPP‐MM‐S hydrogel caused the most well‐structured manner of regeneration, whereas in the absence of metformin, only a slight enhancement in regeneration occurred (Wang, Chang, et al. 2023).

Concerning the medications with antibacterial activity against periodontal pathogens, in a study by Liu et al. (2021), a novel thermosensitive hydrogel was designed containing FPM. This protein was utilized as an anchor protein for SAMP, which avoids its release into the environment (Liu et al. 2021). Besides, FPM is known to be susceptible to a specific virulence factor secreted by *P. gingivalis*, called gingipain. As soon as the FPM of the hydrogel was cleaved by gingipain, the SAMP could be released into the environment to inhibit the vitality of *P. gingivalis* (Liu et al. 2021). The application of antibacterial peptides against periodontal pathogens can be a viable solution to circumvent the multi‐antibiotic resistance of several bacterial species (Enigk et al. 2020). Some of these peptides are involved in innate immune responses in the saliva, especially gingival defensins and cathelicidins that could be used as potential biomarkers to detect early periodontitis (Güncü et al. 2015). Moreover, a study by Enigk et al. (2020) on the antibacterial activity of these peptides against periodontal pathogens has indicated that nisin (produced by *Lactobacillus lactis*) (Kuipers et al. 1995), melittin (the main component of honey bee venom) (Higgins and GSe 2011), and lactoferrin (present in saliva) (Vorland 1999) can be potential candidates to effectively eliminate periodontal pathogens. These peptides could further be utilized in thermosensitive hydrogels to develop a more potent antibacterial activity.

Among the studies applying thermosensitive hydrogels to regenerate the periodontal tissue, only three studies have investigated the efficacy of loading stem cells on these biomaterials. In the study by Chien et al. (2018), iPSCs were utilized in a CS/gelatin/β‐GP hydrogel. The results indicated that the combined application of iPSCs (2 × 10^4^ cells/20 μL hydrogel) and BMP‐6 (1 ng/mL) could significantly enhance the expression of genes related to osteogenesis; besides, this combination achieved the highest rate of new bone volume, trabecular number, and trabecular thickness based on μ‐CT results (Chien et al. 2018). According to the histology examinations, the hydrogel‐iPSCs‐BMP‐6 was able to regenerate cementum and PDL, as well as bone and connective tissue (Chien et al. 2018). Besides the regenerative potentials, iPSCs could also modulate the degree of inflammation by reducing the number of inflammatory cells and cytokines (IL‐1β, IL‐8, and TNF‐α). This phenomenon can be helpful because the reduction of inflammation in the grafted area can reduce the risk of graft rejection. In another study by Ammar et al. (2018), the viability of PDLSCs encapsulated in CS/HTCC/β‐GP was assessed. The results indicated that the addition of 10–15 mg/mL FDPC could significantly enhance the survival rate of PDLSCs during 7 days (Ammar et al. 2018). In another study by Pan, Deng, et al. (2019), PDLSCs underwent lentiviral transduction resulting in overexpression of PDGF‐BB. The modified PDLSCs were loaded on a PLGA–PEG–PLGA thermosensitive hydrogel. The cell viability analysis demonstrated satisfactory biocompatibility of PLGA–PEG–PLGA hydrogel during the 7 days of the experiment. The in vivo outcomes also demonstrated that the encapsulation of PDLSCs overexpressing PDGF‐BB in the mentioned hydrogel exhibited the highest amount of bone regeneration compared to the negative control, hydrogel alone, and hydrogel loaded with PDLSCs without overexpression of PDGF‐BB (Pan, Deng, et al. 2019). These results were also confirmed in histological assessment and ALP staining demonstrating that hydrogel loaded with PDLSCs overexpressing PDGF‐BB could achieve the most satisfactory outcomes.

Overall, it was indicated that the involvement of bioactive agents and medications can result in significant antibacterial (Liu et al. 2021), anti‐inflammatory (Xu et al. 2019; Liu et al. 2022), and regenerative outcomes (Ammar et al. 2018; Xu et al. 2019; Chien et al. 2018) (Figure 2). Moreover, the addition of stem cells can highly potentiate bone regeneration (Chien et al. 2018; Pan, Deng, et al. 2019) and decrease inflammation (Chien et al. 2018).

## 8 Insights Into Clinical Practice

Based on this systematic review, thermosensitive hydrogels can be a potential option in periodontal tissue regeneration. These biomaterials could be considered effective biomaterials with desired properties that can turn into the gel state in the body temperature. To optimize the thermosensitive characteristics of these hydrogels for clinical applications, the gelation time and the gelation temperature are important. The gelation time of thermosensitive hydrogels, when placed at the desired temperature (37°C), varied from 3 min to even 20 min (Table 3) based on the type of used biomaterials and the relative ratios when combined. The shortest gelation time achieved in the study by Wang, Peng, et al. (2023) was 40 s at 37°C, which is practical for periodontists to apply in clinical settings because of the possibility of saving time and avoiding patient discomfort.

**Table 3:** Representation of the polymer and thermogelling time of thermosensitive hydrogels for periodontal regeneration.

Author (year)	Thermosensitive polymer	Thermogelling time (min)/temperature (°C)
Huang et al. (2023)	I. PVA (0%)/CS II. PVA (1%)/CS III. PVA (2%)/CS IV. PVA (4%)/CS	NR
Tan et al. (2023)	I. CS/β‐GP/β‐TCP (0%) II. CS/β‐GP/β‐TCP (2%) III. CS/β‐GP/β‐TCP (4%) IV. CS/β‐GP/β‐TCP (6%)	NR
Wang, Zhang, et al. (2023)	CS/Gelatin/β‐glutamine/T8IC/BMP‐2	2 min/45°C
Wang, Peng, et al. (2023)	CS/β‐GP/SA	3 min/37°C
Wang, Chang, et al. (2023)	PDLLA‐PEG‐PDLLA‐Met@MSN‐SDF‐1	NR
Wang, Peng, et al. (2023)	FA‐GEN‐Lip@Gel	40 s/37°C
Liu et al. (2022)	PEGDA	5 min/37°C
Arpornmaeklong et al. (2021)	I. Chitosan/β‐GP II. Collagen/β‐GP III. Chitosan/β‐GP/Collagen‐β‐GP (2:1) IV. Chitosan/β‐GP/Collagen‐β‐GP (1:1)	I. 10 min/37°C II. 20 min/37°C III. 15 min/37°C IV. 10 min/37°C
Liu et al. (2021)	PEGPD@SDF‐1	10 min/37°C
Petit et al. (2020)	CS/GP	NR
Pan, Deng, et al.^a^ (2019)	I. PLGA/PEG/PLGA (15%) II. PLGA/PEG/PLGA (20%) III. PLGA/PEG/PLGA (25%) IV. PLGA/PEG/PLGA (30%)	NR
Xu et al. (2019)	CS/β‐GP/gelatin	5 min/37°C
Zang, Mu, et al. (2019)	Chitosan/β‐GP	NR
Ammar et al. (2018)	Chitosan/β‐GP	10 min/37°C
Chien et al. (2018)	Chitosan/Gelatin/GP	NR
Li, Pan, et al. (2016)	I. CS/GP II. CS/CSn/pDNA‐BMP2/GP	I. 5 min/37°C II. 3 min/37°C
Li, Ji, et al. (2017)	I. CS/GP II. CS/CSn/pDNA‐BMP2/GP	I. 1 min/37°C II. 1 min/37°C
Zang et al. (2014)	I. CS‐SA/β‐GP (9:1) II. CS‐PA/β‐GP (9:1) III. CS‐PA/β‐GP (7:1)	I. 30 min/37°C II. 5–6 min/37°C III. 5–6 min/37°C
Ji et al.^b^ (2010)	I. CS/HTCC/α,β‐GP (8.33 v/v%) II. CS/HTCC/α,β‐GP (9.09 v/v%)	I. 3 min/37°C–10 min/35°C II. 3 min/37°C–3 min/35°C

Abbreviations: CS, chitosan; CS‐PA, autoclaved chitosan powder; CS‐SA, autoclaved chitosan solution; CSn, chitosan nanoparticle; SA, sodium alginate.

^a^ Only the sol–gel transition temperature for PLGA/PEG/PLGA (20%) was reported, which was 34.33 ± 0.57°C.

^b^ The study indicated that the sol–gel transition occurred at 25°C. The gelation times for 30°C and 25°C are not reported in Table 2.

Although the body temperature is 37°C, the temperature of the periodontal tissue is not necessarily the same. Studies assessing the periodontal tissue temperature are scarce. However, a study by Ng, Compton, and Walker (1978) found that in an ambient environment temperature of 22.8 ± 0.2°C, the temperature of the sulcus of individuals with a healthy periodontium was 33.9 ± 0.4°C. It is worth mentioning that in periodontitis, due to higher inflammation status, the temperature rises; however, the highest reported temperature caused by periodontitis is around 35°C, which can considerably affect the gelation time (Ji et al. 2010). Moreover, the mechanical strength and degradation rate of the hydrogels should also be considered when optimizing their clinical applicability of thermosensitive hydrogels. The mechanical strength can be modulated through the degree of polymers cross‐linking (Jahan, Mathad, and Farheen 2016) or the addition of osteoinductive and osteoconductive bioceramics, including hydroxyapatite (Xiang et al. 2022; Suvarnapathaki et al. 2020). Because the oral cavity is a dynamic environment with various forces applied in different directions (Duanmu et al. 2021), the accurate design of thermosensitive hydrogels to withstand these forces can predict the success rate of the treatment. Moreover, the degradability of the scaffold is important to be adjusted. In an ideal condition, the degradation of the hydrogel should be equal to the rate of tissue regeneration (Amiri, Lavaee, and Danesteh 2022). The faster degradation of the hydrogel would considerably deprive the body tissue of a proper environment to regenerate the defect (Amiri, Lavaee, and Danesteh 2022). According to the current literature (Ammar et al. 2018; Xu et al. 2019; Chien et al. 2018; Ji et al. 2010; Liu et al. 2021; Arpornmaeklong et al. 2021; Pan, Deng, et al. 2019; Zang et al. 2014; Liu et al. 2022; Huang et al. 2023), more studies are required to optimize the in vitro and clinical characteristics of thermosensitive hydrogels. Moreover, further studies with clinical evaluation of these biomaterials are necessary to validate their potential in the clinical setting.

## 9 Conclusions

The application of thermosensitive hydrogels presents a novel and promising avenue for periodontal regeneration, offering a versatile platform that can conform to defects, sustain the release of therapeutic agents, and create bioactive microenvironments conducive to tissue repair. By incorporating bioactive agents, medications, and stem cells, these hydrogels show potential for enhancing anti‐inflammatory and regenerative effects in periodontal regeneration. Future research should focus on optimizing the in vitro and clinical characteristics of thermosensitive hydrogels, as well as conducting further studies to validate their efficacy in clinical settings. Additionally, designing thermosensitive hydrogels to withstand dynamic forces and adjusting their degradation rates to match tissue regeneration could be crucial for the success of future treatments.

## Author Contributions

M.A.A. devised the idea and developed the study design. M.A.A. and D.A. performed the data extraction and wrote the original draft. S.H. critically revised and validated the study. All authors have read and approved the final manuscript.

## Ethics Statement

The authors have nothing to report.

## Consent

The authors have nothing to report.

## Conflicts of Interest

The authors declare no conflicts of interest.

## Data Availability

The data are available by the corresponding authors upon a reasonable request.
